# Stapler-less burst pressure in an ex vivo human gastric tissue: a randomized controlled trial

**DOI:** 10.1007/s13304-021-00975-y

**Published:** 2021-01-26

**Authors:** Gianmattia del Genio, Claudio Gambardella, Salvatore Tolone, Luigi Brusciano, Domenico Parmeggiani, Mariachiara Lanza Volpe, Francesco Saverio Lucido, Ludovico Docimo

**Affiliations:** Division of General, Mininvasive and Bariatric Surgery, University of Campania “Luigi Vanvitelli”, Via Pansini 5, 80100 Naples, Italy

**Keywords:** Sleeve gastrectomy, Stapler-less, Burst pressure, Leaks, Manometry

## Abstract

Stapler-less laparoscopic sleeve gastrectomy (LSG) is emerging as a new potential affordable cost-effective alternative procedure. However, no pre-clinical data are currently available on human tissue. We aimed to evaluate whether traditionally suturing without the use of surgical stapling may produce a comparable bursting pressure on human gastric tissue. A prospective cohort of consecutive patients undergoing LSG was divided in two groups to compare a barbed extra-mucosal running suture (stapler-less) versus a standard stapler line. A burst pressure test was applied to the gastric specimen employing high-resolution manometric catheter. Type, location and features of the leak were described. We enrolled a total of 40 obese patients, 20 patients for each group. Median burst pressures of the stapler-less group resulted statistically significant increased (*p* < 0.0001) than the one in standard stapler group. In all cases, leak occurred along the surgical closure site independently from the used technique (group 1 vs 2; *p* = N.S.), more often at the proximal stomach (*p* < 0.05). In human ex vivo model, traditional surgical suture (i.e. running hand-sewn) produced an effective temporary closure, with superior resistance to increasing volume and pressure. How this may impact on clinical LSG outcomes needs further evaluations and was not the object of this study.

**Supplementary Information:**

The online version contains supplementary material available at 10.1007/s13304-021-00975-y.

## Introduction

Given the rising rate of morbid obesity and associated comorbidities with increasing financial pressures on healthcare systems worldwide, alternative ways to carry out cost-effective laparoscopic sleeve gastrectomy (LSG) are advocated [1, 2].

Recently, staple line reinforcement by oversewing or buttressing with various materials seems to reduce the incidence of leak and bleeding [3–5].

That is, some authors reported limited initial reports of stapler-less sleeve gastrectomy created via energy-based resection and closed by sutures alone [6]. However, due to the lack of data, the bariatric community remains sceptical about stapler-less procedures, considered cumbersome and time-consuming, and currently far from being a valid alternative [7].

In the attempt to investigate and provide useful data for the bariatric community, we designed an ex vivo study to evaluate human gastric tissue resistance following standard stapling and traditional hand-sewn running closure. Limitation and criticism of such approach are largely discussed.

## Methods

### Study design and cohort

A prospective, open-labelled, randomized controlled trial was conducted to evaluate the feasibility of stapler-less sleeve gastrectomy in an ex vivo model. Our institutional review board approved the study protocol. The study protocol was registered on ClinicalTrials.gov (Identifier: NCT04488042). We adhered to the CONSORT guidelines in reporting this trial’s results.

We prospectively enrolled a consecutive series of 40 patients underwent LSG at the Division of General, Mini-Invasive and Obesity Surgery, of an University Hospital, between January 2020 and June 2020. Each patient was informed about the investigational nature of the study and received detailed information about the study protocol. Before subjects entered the study, specific informed consent was obtained from each.

Subject inclusion and preoperative workup were accomplished according to the Italian society of bariatric and metabolic surgery (S.I.C.O.B.) recommendation, as previously described [8]. Gastric specimen with injury and/or electrocoagulation signs at serosa were excluded from the study and not analysed. Presence of comorbidities capable of affecting specimen tissue resistance (i.e. type II diabetes, gastric ulcer, connective tissue disease, systemic sclerosis, polymyositis) was also considered exclusion criteria.

The extraction of all resected stomach occurred by an operative trocar site, in the attempt to avoid specimen lesion; all ex vivo specimens were analysed immediately after extraction.

### Randomization

Enrollment and randomization were carried out by co-investigators. Participants were randomly allocated to one of the two groups using computer-generated permuted blocks (1:1) (www.randomization.com). Burst pressure test performed by surgeons blinded for group membership and surgical findings. The cohort was randomly divided in two groups as follows: group 1 (stapler-less, hand-sewn) and group 2 (no reinforcement).

### Study intervention

**Fig. 1:**
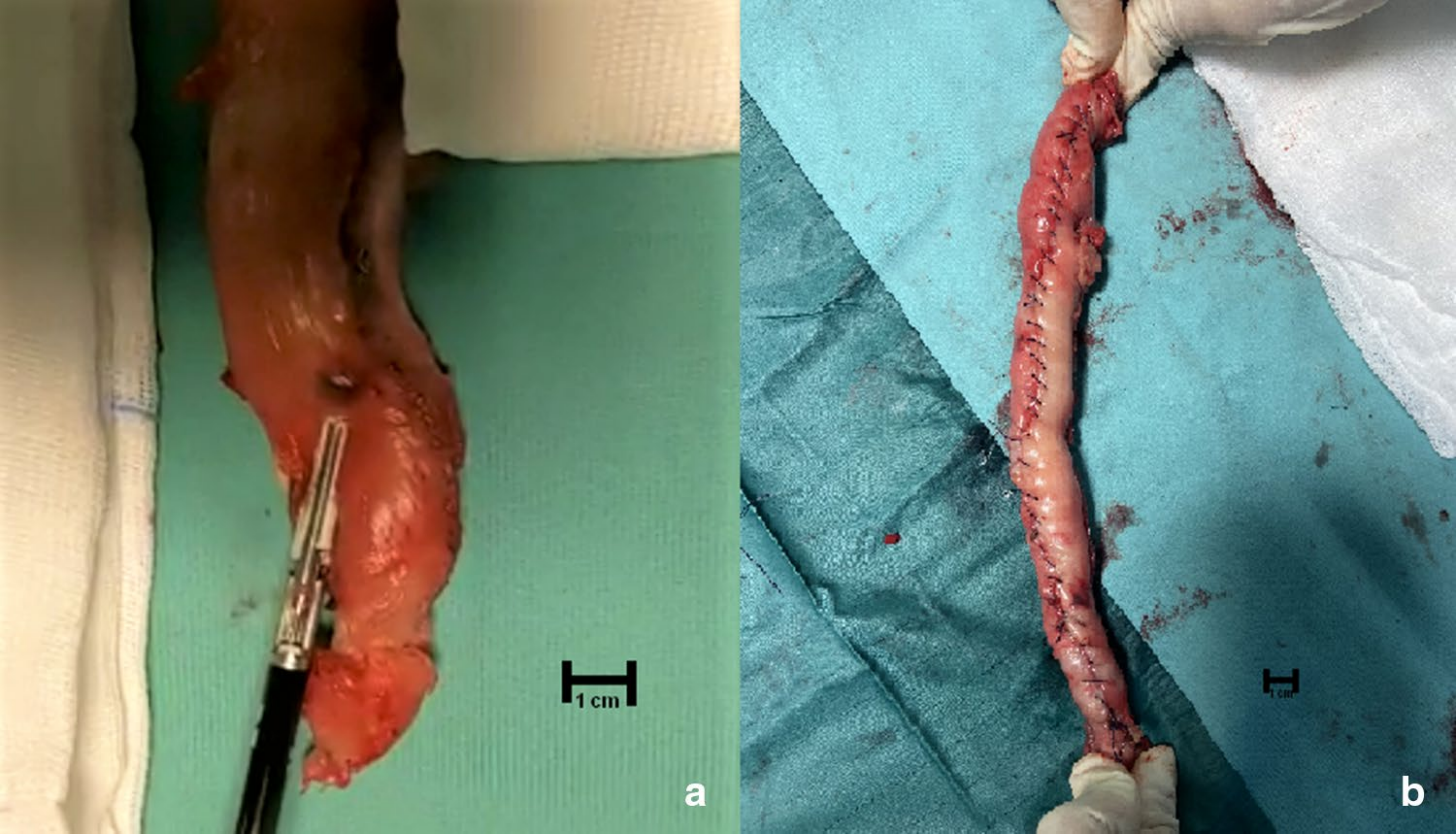
Gastric specimen in stapler-less group. **a** Stapler line removal by electrothermal bipolar-activated device (LigaSure Atlas™, Valleylab, Boulder, CO, USA). **b** A stapler-less hand-sewn reconstruction was adopted. A single extra-mucosal running barbed suture (3/0V-Loc™ suture; Covidien, Mansfield, MA, USA), incorporating sero- and submucosal gastric layers, closed the gastric tube

In group 1, after the LSG stapler line removal by electrothermal bipolar-activated device (LigaSure Atlas™, Valleylab, Boulder, CO, USA), a stapler-less hand-sewn reconstruction was adopted along a 40F bougie. A single extra-mucosal running barbed suture (3/0V-Loc™ suture; Covidien, Mansfield, MA, USA), incorporating sero- and submucosal gastric layers, closed the gastric tube. (Fig. 1) In group 2, no reinforcement was performed, the stomach was re-sleeved along a 40F bougie with Echelon Flex Endopath 60-mm linear stapler with gold cartridge (Ethicon Endo-Surgery, Cincinnati, OH, USA) to reproduce standard volume of remnant LSG stomach and/or eliminating zig-zag shape of suture line.

### Manometric bursting test

**Fig. 2:**
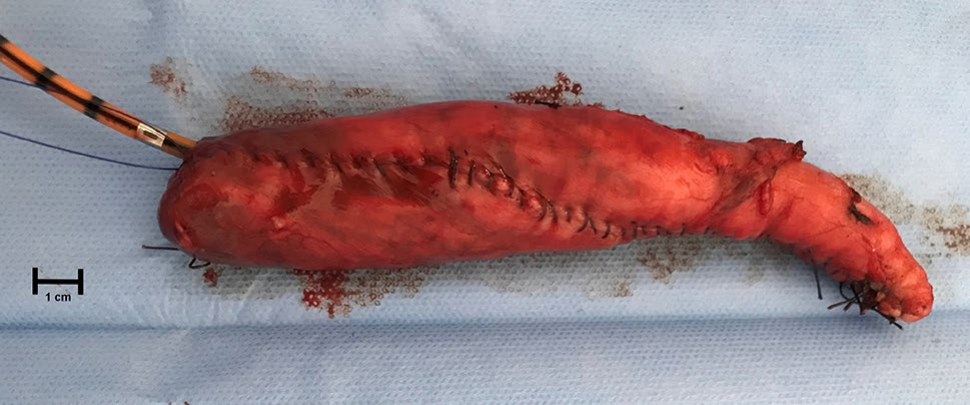
The gastric specimen during burst pressure testing. A high-resolution manometry catheter is placed inside the gastric lumen, and this is filled with a NaCl 0.9% solution and methylene blue. Previously, the gastric specimen is resected to obtain a new linear staple line and a diameter similar to the sleeved stomach

**Fig. 3:**
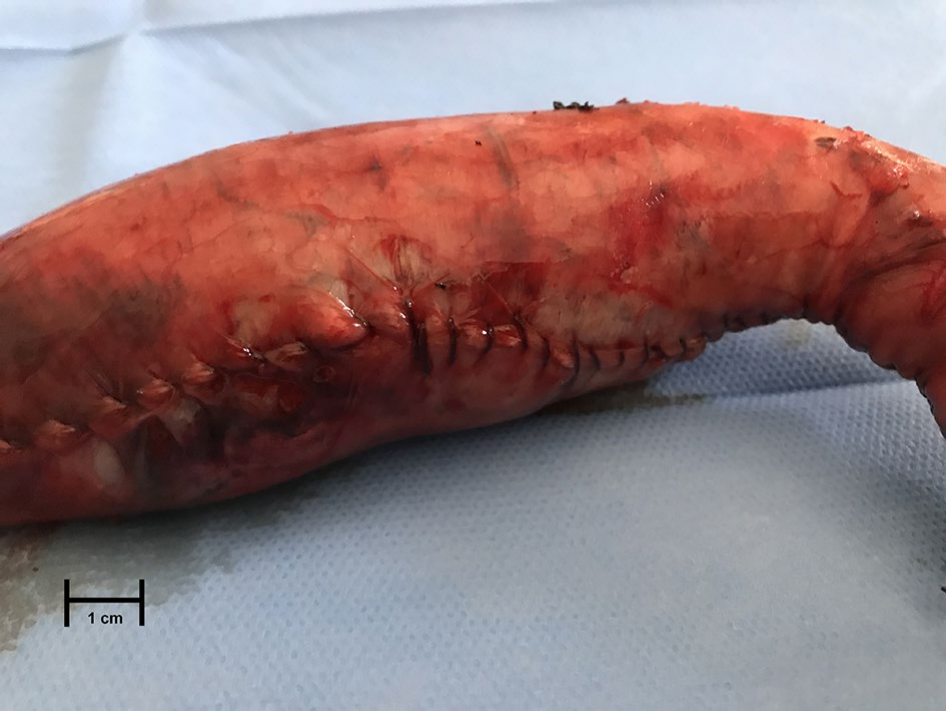
The burst pressure is stopped when a serosal laceration is identified, and methylene blue is seen flowing out through the suture line

Following gastric preparation, burst pressure test was suddenly performed. The manometric test was performed by a blinded physician. A small incision was made at gastric antrum to insert the High-Resolution Anorectal Manometry (HRAM) catheter, tightly fastened. HRAM was performed using a 7 cm long registering sites (Unisensor, Sandhill Sc, Insight g3) solid state probe. A total of 30 pressure sensors, placed apart at five different levels (four radially, one at the probe distal end) constituted the recording system [9, 10]. An electric syringe pump instilled by an open channel into the manometric probe a methylene blue NaCl solution at constant flow (0.5 ml/min); total volume of solution instilled was recorded (Fig. 2). The pressure level was recorded as the burst pressure when the first methylene blue leak was detected (Bioview Analysis software, Sandhill Sci) [9]. Site of leaks along the staple line, according to gastric anatomy (e.g. fundus, body and antrum) was reported (Fig. 3).

### Study outcome

The aim of the study was to evaluate the manometric burst pressure and the saline volume needed to determine a gastric leak in the ex vivo gastric specimen after hand-sewn running suture (group 1), and stapler suturing (group 2), recorded as control case group.

### Statistical analysis

Statistical analysis was performed using SPSS for Windows (version 22; SPSS Inc., Chicago, IL. USA). Continuous data are expressed as median and interquartile (25–75th) range or mean and SD, unless otherwise indicated. For all tests, a two-sided *p* < 0.05 was considered statistically significant. The sample size was calculated setting a power of 0.9 for the quantitative variable (i.e. increasing 50% means in mmHg of bursting pressure for different gastric suturing techniques of the specimen). To reach a significance set at *p* < 0.05 for increasing 50% mean burst pressure, enrolling a minimum of seven patients for each group was needed. Data analysis was conducted according to a per-protocol approach.

## Results

### Study population

**Fig. 4:**
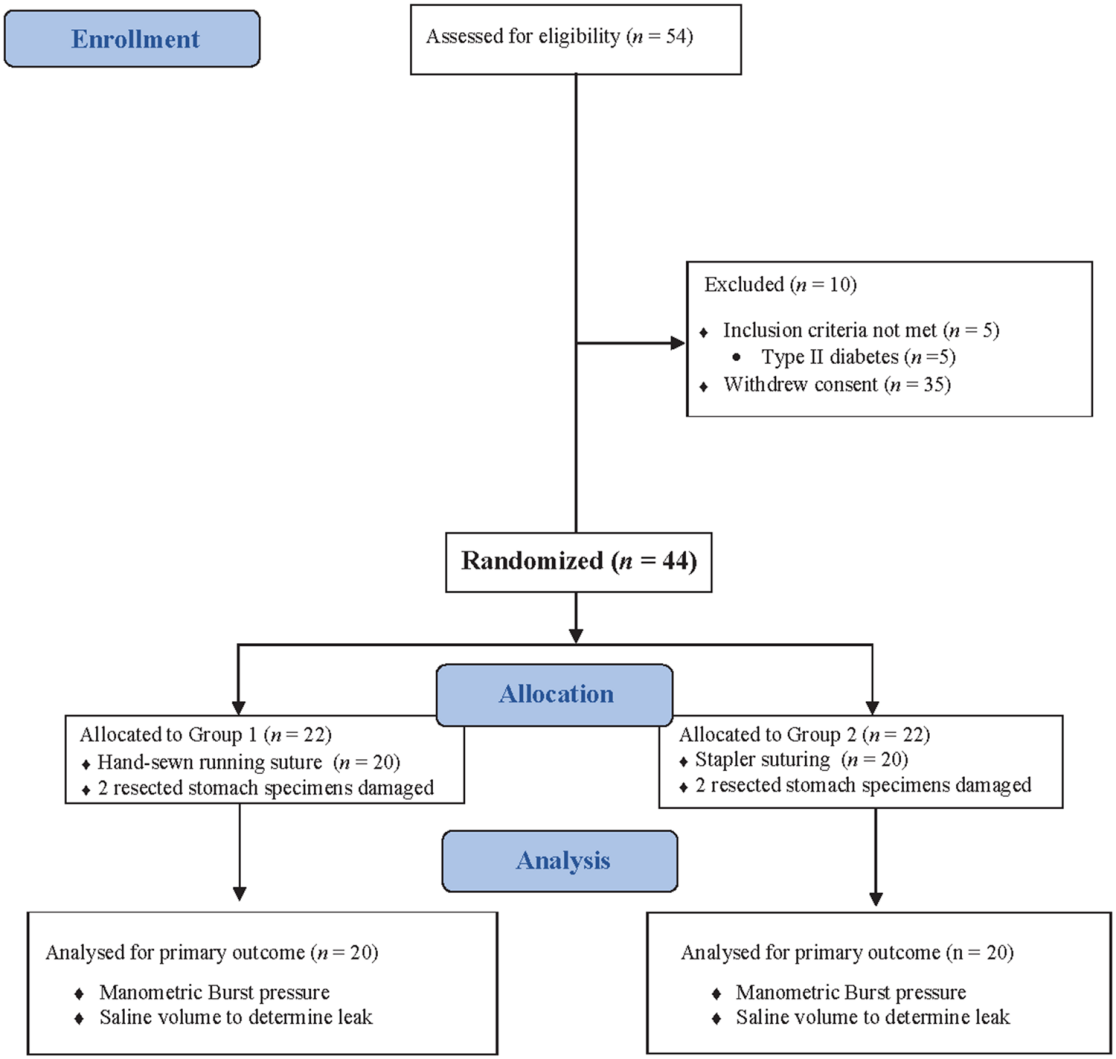
The CONSORT flow diagram. Statistical analysis was performed following a per-protocol approach

Evaluations were performed immediately after each LSG. Between January 2020 and June 2020, a total of 54 morbid obesity patients referred to our institution to perform LSG. 5 patients did not meet inclusion criteria and 5 patients withdrew their consent to participate, whereas 44 met eligibility criteria and were randomly allocated in a 1:1 ratio to receive hand-sewn running suture or stapler closure. Four resected stomach specimens were damaged during LSG and/or extraction from the peritoneal cavity, and, therefore, not included in the cohort. The analysed cohort included 40 obese subjects (24 males, 60%, and 16 females, 133 kg [98–149], BMI = 45 [38–49]), 20 patients underwent hand-sewn running suture (group 1), and 20 cases received stapler suturing (group 2), recorded as control case group. (Fig. 4).

There were no anthropometric (i.e. BMI, sex and age) differences at baseline in the two groups (*p* = N.S.); no patients had preoperative diagnosed type II diabetes, or other comorbidities that may affect the gastric tissue consistency (e.g. ulcer, connective tissue disease, systemic sclerosis, polymyositis) (Table 1).

**Table 1:** Preoperative demographics data of stapler-less (group 1) and stapler group (group 2). BMI (body mass index)

	Stapler-less group (*n* = 20)	Stapler group (*n* = 20)	*p*†
Age	35.2 ± 9*	34.9 ± 7*	0,157
Male	11 (55%)	13 (65%)	0,518
Female	9 (45%)	7 (35%)	0,518
Weight kg	132.9 (98–140)*	133.3 (101–149)*	0,064
BMI	45 (38–47)*	46 (39–49)*	0,4226
ASA(I-II) (%)	13 (65%)	12 (60%)	0,744
ASA(III-IV)(%)	7 (35%)	8 (40%)	0,744
Diabetes mellitus (%)	–	–	–
Chronic obstructive pulmonary disease (%)	2 (2%)	3 (3%)	0,632
Hypertension (%)	12 (60%)	11 (55%)	0,749

*Values are mean ± interquartile range. †Wilcoxon rank sum test for paired data

### Primary outcome

**Fig. 5:**
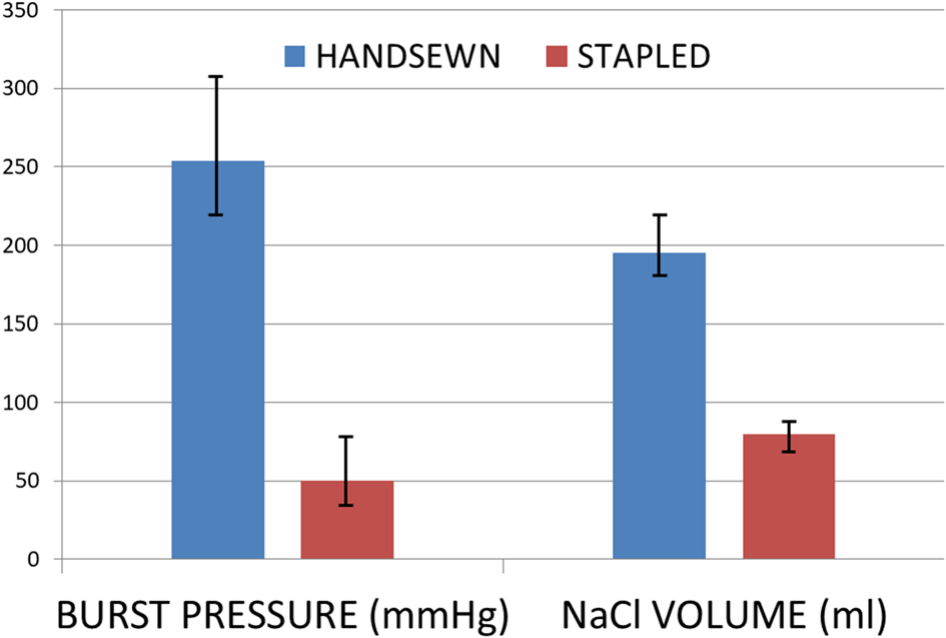
The graph bar comparing burst pressures recorded at high-resolution manometry (expressed in mmHg, *y*-axis) and saline volume (expressed in ml, *y*-axis). The difference between the hand-sewn and the stapled group was statistically significant for both the indicators (*p* < 0.0001)

Mean time for the ex vivo procedure was longer in group 1 (20.4 ± 4.3 min vs 6.05 ± 3.3 min, *p* < 0.05). The sutures (hand-sewn or mechanical) of specimens in both groups were fashioned using a 40F bougie. Median burst pressure was statistically increased in the hand-sewn group (254 [221–313] vs 50 [34–70] mmHg, *group 1 vs 2, respectively*; *p* < 0.0001), with an approximately fivefold value. The volume of saline needed to cause leakage was threefold in group 1 than control group (195 [182–220] vs 80 [70–90] ml, *group 1 vs 2, respectively*; *p* < 0.0001) (Fig. 5).

Location of leaks was always at the suture line; in group 1, localized at the proximal 5 cm segment of gastric remnant in 16/20, at gastric body in 2/20 and at the distal 5 cm in 2/20; in groups 2, leak site was at proximal 5 cm in almost all cases (19/20); one occurred between cut-edges of two linear cartridges.

## Discussion

Sleeve gastrectomy as a single procedure was developed in the era of laparoscopy and gained worldwide popularity in a relatively recent period. Therefore, surgical stapling is currently considered the only way to proceed, especially causes a long gastric transection is required [11]. This was supported by associated advantages, such as standardized resection and closure, with reduced time and challenge for surgeons. Moreover, staplers allow satisfactory bleeding control and closed transection, limiting peritoneal contamination [12].

Not surprisingly, exploring the possibility of obtaining similar results by mean of traditional standardized suturing methods, potentially reducing cost for healthcare systems and expanding surgical offer in emerging countries, is considered cumbersome, time-consuming and still not supported by evidence.

Given the low complication rate currently associated with LSG, a direct comparison of stapled vs traditional hand-sewn is practically impossible requiring several thousand cases to obtain a statistically significant difference [13].

In a recent animal study, Rogula et al. demonstrated the technical feasibility of performing robotic stapler-less gastric resection at the greater curve; authors failed to give a definitive conclusion, being gastric porcine tissue more thick and resistant than human (i.e. no leak at 760 mmHg) [14].

Though several suturing variables might be implied in LSG. Here we have identified the role for suture closure type in contrasting increased volume and pressure with a specific accurate measurement (high-resolution manometry catheter with multiple electronic sensors spanned along the specimen). Indeed, single running barbed closure was able to increase several times (i.e. fivefold) the tissue resistance, compared over the triple-row endostapler. Probably, the continuous suture with an elastic material allows a dynamic tension distribution on a greater surface of the gastric wall and therefore is more effective than metal inelastic staples in the resistance to bursting [2, 3, 14]. How this increase may impact LSG clinical outcomes is far from the aim of this study, being involved many different aspects (e.g. correct approximation of tissue layers, vascularization, suture standardization, thermal injury produced by devices, patient’s healing process capability).

A potential strength of this study lays on the test accomplished immediately after extraction, during the surgical procedure, limiting, as far as possible, the impact of devascularization into the specimen. The “near-the-same” specimen reproduced the volume of the gastric remnant, by re-shaping along the standard bougie (40F), eventually removing any curved “zig-zag” suture line.

A secondary finding was the occurrence of all leaks along the suture line in both group, independently from the technique used. This proves that compared to anatomical tissue, any surgical closure is a potential weak point that may disrupt at a threshold pressure, following a delta increase normally not observed in a physiologic range. Thinner is the tissue (i.e. proximal vs. distal stomach) lower is delta needed to reach the burst pressure level.

In clinical practice, several technical modifications have been advocated and associated to conventional LSG to reduce the onset of postoperative leaks and bleeding, ranging from the staple line oversewing, the adoption of fibrin glue and the experimental use of bovine pericardium, as largely reported [3, 15–17]. In details, several papers already reported the superiority of stapler suture reinforced with running suture in the prevention of postoperative complications, highlighting the importance for tissue tensile strength of the hand-sewn suture itself [3, 15]. Therefore, starting from these encouraging results, in our experimental settings, we adopted the running barbed closure alone evidencing an impressive increase in the gastric tissue resistance; however, differently from the abovementioned methods, we are not able to present data from clinical routine practice.

One limitation of this study is, in fact, the lack of a direct in vivo comparison of LSG vs stapler-less SG, in the setting of a randomized trial, being stapled LSG in authors believe currently the standard of treatment; thus, no conclusion should be extrapolated for adopting hand-sewn closure from this study alone. Therefore, our ex vivo model was only possible to analyze the tensile strength of the resected specimen as consequence to the burst pressure stress; nevertheless, it was not possible to consider and assess all the physiological phenomena involved in “in vivo” tissue healing.

At present, it seems reasonable to assert that manual surgical closure (i.e. running closure) produce in human ex vivo model an effective temporary resistance at a higher level of volume and pressure. If this effect will produce clinical comparable results needs further evaluations and was not the object of this study.

## Supplementary Information

Below is the link to the electronic supplementary material.

Supplementary file1 (DOCX 36 KB)

Supplementary file2 (DOCX 15 KB)

## Data Availability

The datasets used and/or analyzed during the current study are available on reasonable request.
