# Supplementary material for: Stapler-less burst pressure in an ex vivo human gastric tissue: a randomized controlled trial
Source: Updates Surg. 2021 Jan 26;73(2):679–85. doi: 10.1007/s13304-021-00975-y (PMC8005392; doi:10.1007/s13304-021-00975-y)
Supplement: Supplementary file 2 — Supplementary file2 (DOCX 15 KB) [file 13304_2021_975_MOESM2_ESM.docx]

Dear Editor,

here enclosed please find our manuscript entitled

“**Stapler-less burst pressure in a ex-vivo human gastric tissue: a randomized controlled trial**” by Gianmattia del Genio, MD, PhD, FACS, Claudio Gambardella, MD, PhD, Salvatore Tolone, MD, PhD, Luigi Brusciano, MD, PhD, Domenico Parmeggiani, MD, Maria Chiara Lanza Volpe, MD, Francesco Saverio Lucido, MD, Ludovico Docimo, MD.

that we submit for peer review in ***Updates in Surgery***.

All authors have read and complied with author guidelines

All authors contributed significantly to the present research and reviewed the entire manuscript.

The results presented in this paper have not been published previously in whole or part and are not under consideration for publication elsewhere.

One of the major complications after laparoscopic sleeve gastrectomy is represented by leaks along the staple line. Several reinforcement techniques have been proposed, but scarce data about the real strengthening offered are present. Staple line reinforcement by oversewing or buttressing with various materials seems to reduce the incidence of leak and bleeding. Some authors reported limited initial cases of stapler-less sleeve gastrectomy created via energy-based resection and closed by sutures alone. we designed an ex-vivo study to evaluate human gastric tissue resistance following standard stapling and traditional hand-sewn running closure. All were evaluated via burst pressure test by means of high-resolution manometric catheter applied to the gastric specimen. In human ex-vivo model, traditional surgical suture (i.e. running hand-sewn) produced an effective temporary closure, with superior resistance to increasing volume and pressure. How this may impact on clinical LSG outcomes needs further evaluations and was not the object of this study.

We believe that this paper fully falls into the scope and aims of the journal.

We have no financial interest in relation to this paper.

Thank you very much for considering our work for in ***Updates in Surgery.***

Kind regard

Claudio Gambardella
